# Enhanced production of ectoine from methane using metabolically engineered *Methylomicrobium alcaliphilum* 20Z

**DOI:** 10.1186/s13068-022-02104-2

**Published:** 2022-01-13

**Authors:** Sukhyeong Cho, Yun Seo Lee, Hanyu Chai, Sang Eun Lim, Jeong Geol Na, Jinwon Lee

**Affiliations:** 1 C1 Gas Refinery R&D Center, Sogang University, Seoul, Republic of Korea; 2 Department of Chemical and Biomolecular Engineering, Sogang University, Seoul, Republic of Korea

**Keywords:** Methanotroph, Methane, Ectoine, Ectoine biosynthesis pathway, *Methylomicrobium alcaliphilum* 20Z

## Abstract

**Background:**

Ectoine (1,3,4,5-tetrahydro-2-methyl-4-pyrimidinecarboxylic acid) is an attractive compatible solute because of its wide industrial applications. Previous studies on the microbial production of ectoine have focused on sugar fermentation. Alternatively, methane can be used as an inexpensive and abundant resource for ectoine production by using the halophilic methanotroph, *Methylomicrobium alcaliphilum* 20Z. However, there are some limitations, including the low production of ectoine from methane and the limited tools for the genetic manipulation of methanotrophs to facilitate their use as industrial strains.

**Results:**

We constructed *M. alcaliphilum* 20ZDP with a high conjugation efficiency and stability of the episomal plasmid by the removal of its native plasmid. To improve the ectoine production in *M. alcaliphilum* 20Z from methane, the *ectD* (encoding ectoine hydroxylase) and *ectR* (transcription repressor of the *ectABC-ask* operon) were deleted to reduce the formation of by-products (such as hydroxyectoine) and induce ectoine production. When the double mutant was batch cultured with methane, ectoine production was enhanced 1.6-fold compared to that obtained with *M. alcaliphilum* 20ZDP (45.58 mg/L vs. 27.26 mg/L) without growth inhibition. Notably, a maximum titer of 142.32 mg/L was reached by the use of an optimized medium for ectoine production containing 6% NaCl and 0.05 μM of tungsten without hydroxyectoine production. This result demonstrates the highest ectoine production from methane to date.

**Conclusions:**

Ectoine production was significantly enhanced by the disruption of the *ectD* and *ectR* genes in *M. alcaliphilum* 20Z under optimized conditions favoring ectoine accumulation. We demonstrated effective genetic engineering in a methanotrophic bacterium, with enhanced production of ectoine from methane as the sole carbon source. This study suggests a potentially transformational path to commercial sugar-based ectoine production.

**Graphical Abstract:**

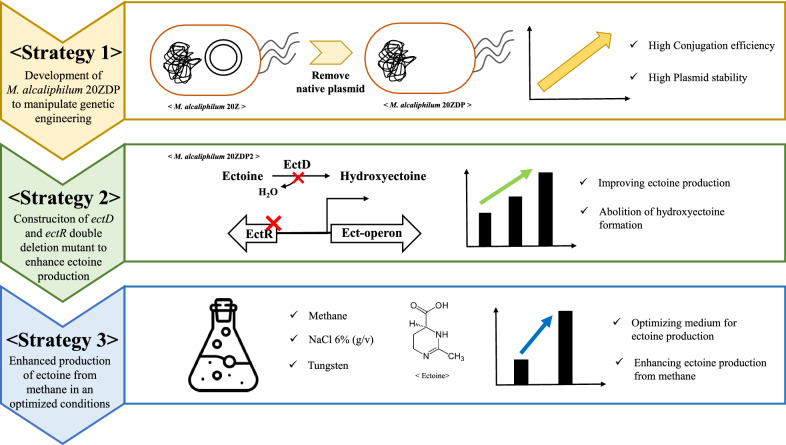

**Supplementary Information:**

The online version contains supplementary material available at 10.1186/s13068-022-02104-2.

## Background

Methane, a greenhouse gas that is 20 times more potent than carbon dioxide, is an energy-rich, inexpensive, and abundant carbon source [1–3]. Methane constitutes more than 80% of natural gas and is a major component of biogas from landfills and anaerobic fermentation [1, 4, 5]. More than 500 million tons of methane gas is generated every year, and this amount is gradually increasing. However, emission reduction through physico-chemical techniques is inefficient and expensive because of the high-temperature and high-pressure conditions. As an alternative, many studies have suggested that the biological conversion of methane can replace physico-chemical methods by combining the conversion of diluted methane emissions with the production of high-value products as a low-cost and environmentally efficient method to mitigate climate change [6–8].

Methanotrophs are bacteria that utilize methane as their sole source of carbon and energy and are promising industrial biocatalysts for the bioconversion of methane to value-added chemicals and fuels [2, 9]. The bioconversion of methane to value-added products (such as single cell proteins, biopolymers, and lipids) using aerobic methanotrophs has been studied for decades [2]. Recently, new methanotrophs have been isolated, and current biological engineering approaches have provided new opportunities for the development of industrial methanotroph strains [10–12]. Despite these efforts, it is necessary to overcome shortcomings such as their low growth rate, the limited genetic tools for their manipulation, and the insufficient fundamental knowledge for using methanotrophs as industrial strains [13].

Ectoine is a compatible solute produced by halophilic and halotolerant microorganisms, such as those from the genera *Halorhodospira*, *Halomonas*, *Chromohalobacter*, and *Vibrio* [14–16]. This organic solute can be synthesized de novo or taken up from a moderately hypersaline environment. It is commonly used as an active ingredient in skin care and sunscreen products to stabilize proteins and other cell structures, and has a wide range of applications in biomedicine [17, 18]. Commercially, *Halomonas elongata*, a moderately halophilic bacterium, is a commonly used ectoine producer that has an established biosynthetic pathway for ectoine metabolism using glucose as a carbon source [19, 20]. Several reports considered to the heterologous synthesis of ectoine in a well-established industrial host such as *Escherichia coli* and *Corynebacterium glutamicum* [21, 22].

**Fig. 1:**
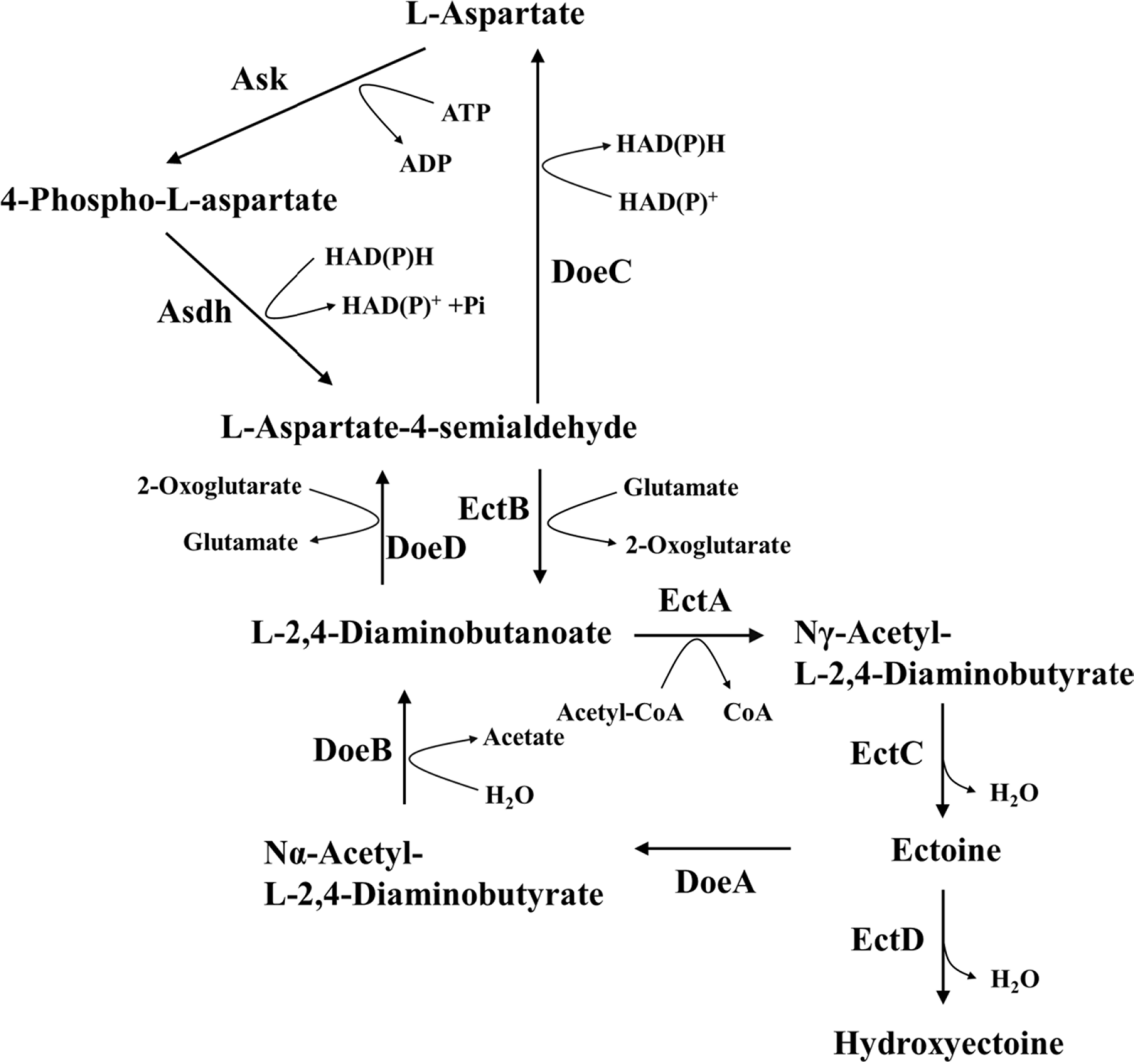
Metabolic pathway of ectoine in *M. alcaliphilum* 20Z. *Ask* aspartate kinase, *AsdH* b-aspartate-semialdehyde-dehydrogenase, *EctB* l-2,4-diaminobutyrate transaminase, *EctA* l-2,4-diaminobutyrateNγ-acetyltransferase, *EctC* ectoine synthase, *EctD* ectoine hydroxylase, *DoeA* ectoine hydrolase, *DoeB* *N*α-acetyl-l-2,4-diaminobutyrate deacetylase, *DoeD* l-2,4-diaminobutyrate transaminase, *DoeC* aspartate-semialdehyde dehydrogenase, *Ask* aspartate

Recently, several studies have proposed the bioconversion of methane to ectoine by the methanotrophic ectoine-producing strain *M. alcaliphilum* 20Z. Thus, the treatment of diluted methane emissions coupled with the synthesis of ectoine in suspended growth bioreactors could potentially reduce the costs associated with ectoine production while boosting climate change mitigation via active methane abatement [23, 24]. The synthesis of ectoine in *M. alcaliphilum* 20Z proceeds from l-aspartate-β-semialdehyde and is catalyzed by the sequential action of l-2,4-diaminobutyrate transaminase (EctB), 2,4-diamino acetyltransferase (EctA), and ectoine synthase (EctC) (Fig. 1) [25, 26]. Hydroxyectoine is directly synthesized from ectoine by ectoine hydroxylase (EctD) (Fig. 1). In *M. alcaliphilum* 20Z, the ectoine biosynthetic genes were organized in the ectABC-ask operon which also contained the additional ask gene, encoding aspartokinase [25, 26]. The transcriptional regulation mechanisms of these ectoine biosynthetic genes in *M. alcaliphilum* 20Z were identified and described, and the MarR-like transcriptional regulator (EctR) was found to suppress the expression of ectABC-ask operon by binding to the putative—10 sequence [27].

In this study, we confirmed that removal of the native plasmid in *M. alcaliphilum* 20Z increased both the conjugation efficiency and the stability of replication of the episomal plasmid. In addition, ectoine production was enhanced by the metabolically engineered *M. alcaliphilum* 20Z, in which *ectD* (encoding ectoine hydroxylase) and *ectR* (transcription repressor of *ectABC-ask* operon) were deleted to reduce the formation of the by-products and to induce ectoine production using methane as the sole carbon source. Further enhancement of ectoine production during batch culture was attempted by optimizing the culture conditions such as by adding tungsten, increasing the salinity, and investigating the appropriate growth phase for maximum ectoine production. The results of this study demonstrate the feasibility of using *M. alcaliphilum* 20Z as a promising biocatalyst for ectoine production from methane.

## Results and discussion

### Development of *M. alcaliphilum* 20Z mutant for efficient genetic engineering by the removal of the native plasmid

The process of the genetic manipulation of methanotrophs has not yet been fully established, and this serves as a critical limitation for bioconversion technologies. Although conjugation methods are commonly used to transform methanotrophs, their transformation efficiency is still very low, and the appearance of transconjugants takes a long time. A previous study reported that the loss of the native plasmid increased the conjugation efficiency in *Methylomicrobium buryatense* [28]. *Methylomicrobium alcaliphilum* 20Z also contains several enzymes of the restriction modification system (Type III RM system) and P-type conjugal transfer system, some of which are located on its native 12.8-kb plasmid. Therefore, we constructed a mutant of *M. alcaliphilum* 20Z to improve the transformation efficiency by eliminating its native plasmid. To achieve this, we knocked out the replication gene *repB* of the native plasmid locus by pK19mobsacB using the sucrose counter-selection method [29]. The resulting mutant of *M. alcaliphilum* 20Z was named “*M. alcaliphilum* 20ZDP,” and was confirmed by colony PCR at three different loci (*repB*, *korB*, and *trbF*) on the native plasmid (Additional file 1: Fig. S1). There was no significant difference in cell growth under normal conditions between the *M. alcaliphilum* 20ZDP and *M. alcaliphilum* 20Z wild-type (data not shown), suggesting that the native plasmid does not contain essential genes for growth under normal conditions.

**Fig. 2:**
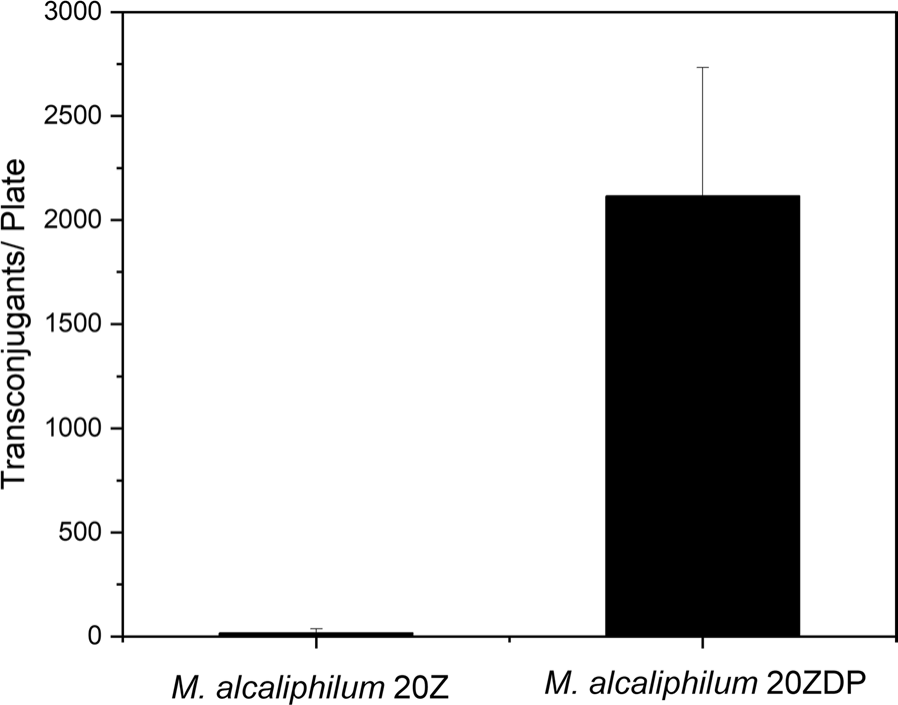
High conjugation frequency of *M. alcaliphilum* 20ZDP by loss of native plasmid. Number of transconjugants containing the small IncP-based plasmid pAWP89 for *M. alcaliphilum* 20Z and *M. alcaliphilum* 20ZDP

To confirm the increase in the transformation efficiency in the mutant strain, *M. alcaliphilum* 20ZDP was manipulated to introduce the episomal vector pAWP89 using the conjugation method. Transformation was carried out five times each on both *M. alcaliphilum* 20Z and *M. alcaliphilum* 20ZDP, resulting in 16 and 2115 colonies per plate after transformation, respectively (Fig. 2). This result showed an increase in the transformation efficiency by approximately 132-fold on average for *M. alcaliphilum* 20ZDP compared to *M. alcaliphilum* 20Z. In addition, there were a number of plates in which no colonies were formed when *M. alcaliphilum* 20Z was used as a conjugant. This result demonstrates that *M. alcaliphilum* 20ZDP is able to replicate IncP-based vectors due to the removal of the native plasmid found in *M. alcaliphilum* 20Z and suggests that there is competition between IncP-based vectors and the native plasmid.

**Fig. 3:**
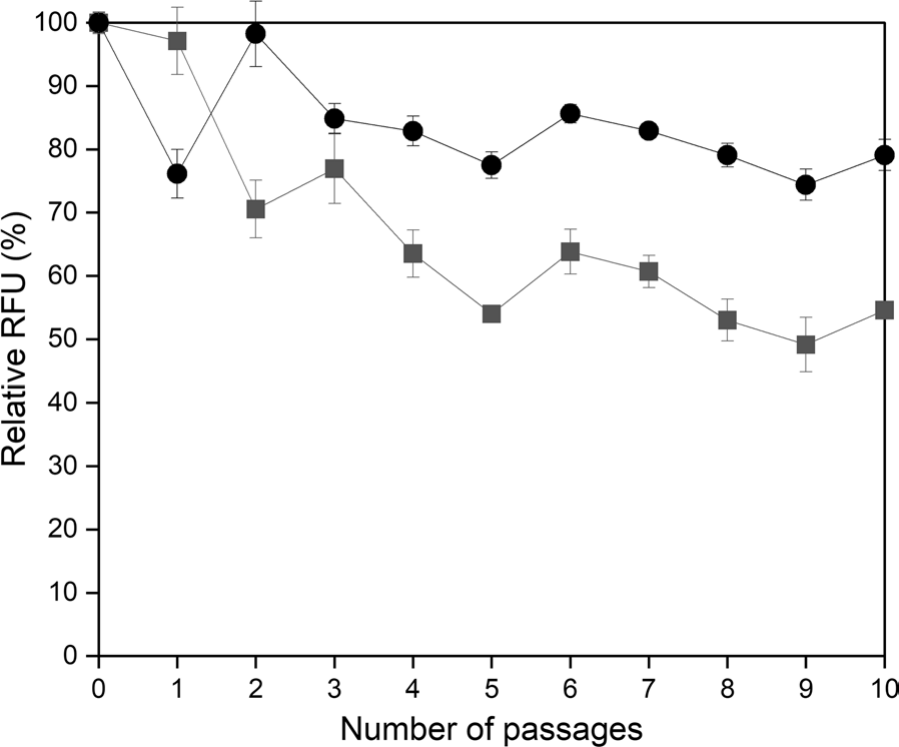
Determination of the stability of the IncP-based plasmid pAWP89. dTomato fluorescence level was measured in the continuous culture of *M. alcaliphilum* 20Z/dt and *M. alcaliphilum* 20ZDP/dt. Symbols indicate *M. alcaliphilum* 20Z/dt (■) and *M. alcaliphilum* 20ZDP/dt (●). All experiments were performed in triplicate and the range of the raw data was within ± 5% of the average

Plasmid-based gene expression systems are useful genetic tools for strain manipulation, and the stability of episomal plasmids in which the desired protein is continuously expressed is very important. To investigate the stability of the episomal plasmid in *M. alcaliphilum* 20ZDP, we examined the enzyme expression level by the introduction of the episomal vector pAWP89 containing the dTomato reporter gene, and determined the extent to which it is maintained after 10 generations. The fluorescence from dTomato expression in *M. alcaliphilum* 20ZDP and *M. alcaliphilum* 20Z was detected using a fluorescence plate reader. As shown in Fig. 3, the dTomato expression levels were similar between *M. alcaliphilum* 20Z/dt and *M. alcaliphilum* 20ZDP/dt in the first generation. After the 10^th^ subculture, the expression level in *M. alcaliphilum* 20ZDP/dt was maintained at 80% of its initial level, while the expression level in *M. alcaliphilum* 20Z/dt decreased by 50%. Therefore, these results indicate that the loss of the native plasmid not only increases conjugation frequency, but also induces stability during the replication of the episomal plasmids.

### Ectoine production of *M. alcaliphilum* 20ZDP in batch culture

In a previous study, *M. alcaliphilum* 20Z was assessed for its ability to produce ectoine using methane as the sole carbon source [24]. The full genome sequence containing the genes involved in ectoine biosynthesis has been investigated in *M. alcaliphilum* 20Z [26]. To evaluate ectoine production by *M. alcaliphilum* 20ZDP, flask batch fermentation was performed in a *Methylomicrobium* medium containing 3% NaCl with methane as the sole carbon source for 72 h. Intracellular and extracellular ectoine and hydroxyectoine were measured as described by a previous method [30]. Intracellular ectoine and hydroxyectoine production by *M. alcaliphilum* 20ZDP was slightly reduced compared to that of the wild-type strain (data not shown). No extracellular ectoine or hydroxyectoine was detected during cultivation under culture conditions. Considering the efficiency of genetic engineering for the improvement of ectoine production, it was decided to use *M. alcaliphilum* 20ZDP as an ectoine producer in a further study.

**Fig. 4:**
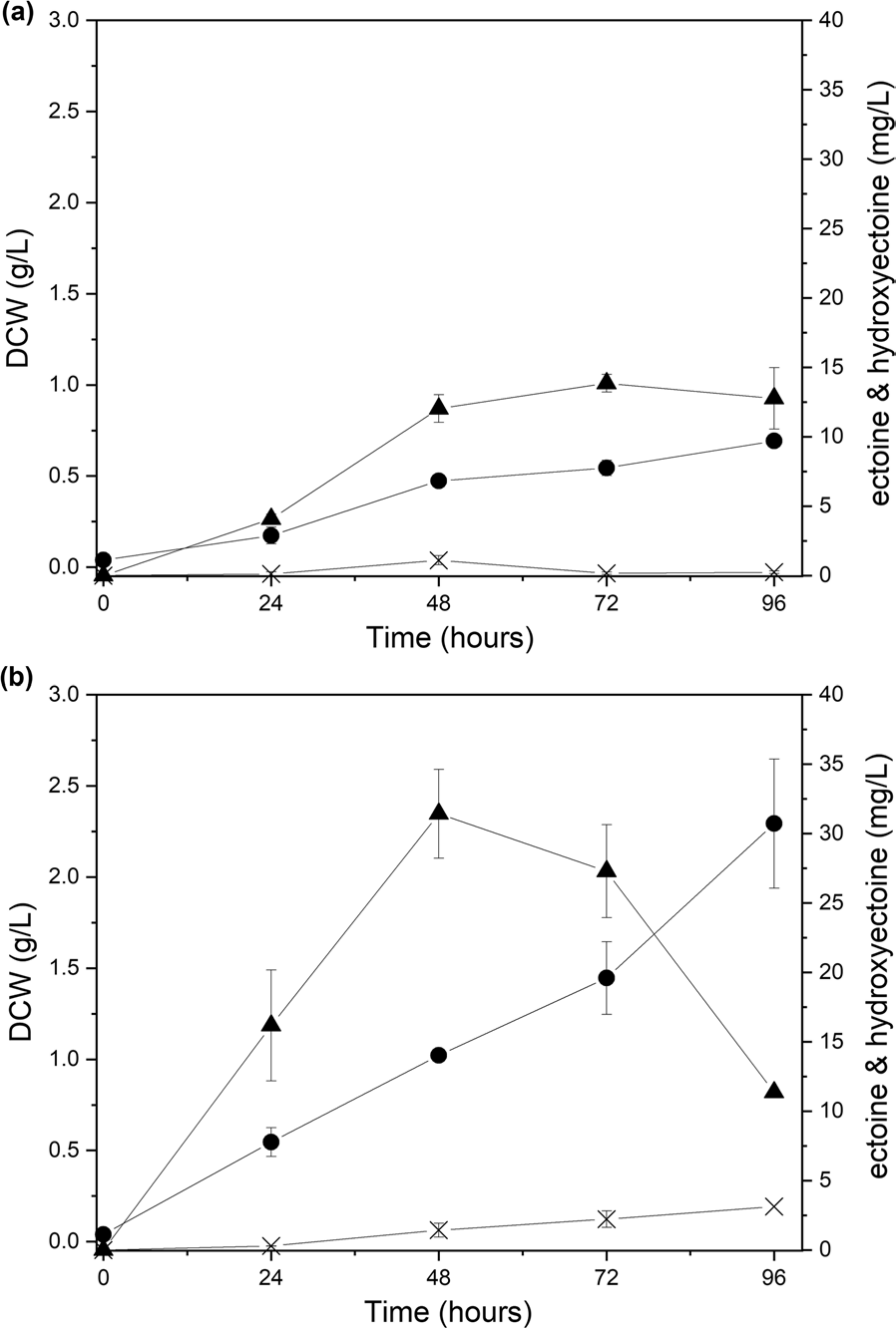
Effect of tungsten (W) addition on cell growth and ectoine production in *M. alcaliphilum* 20ZDP. Time course of the biomass (dry cell weight; DCW), ectoine, and hydroxyectoine production from methane by *M. alcaliphilum* 20ZDP cultivated in W-free medium (**a**) and in W-added medium (**b**), respectively. The following symbols were used: DCW (●), ectoine (▲), and hydroxyectoine (X). All experiments were performed in triplicate and the range of the raw data was within ± 5% of the average

In our previous study, we observed that the growth of *M. alcaliphilum* 20Z in a batch culture, with methane as the sole carbon source, was stimulated by the addition of tungsten [31]. To investigate the effect of tungsten on the increase in biomass and thereby on ectoine production, tungsten (0.05 μM) was added to the *Methylomicrobium* medium containing 3% NaCl, and cell growth and ectoine production were measured. The time-course cultivation profiles of *M. alcaliphilum* 20ZDP in tungsten-added and tungsten-free media are shown in Fig. 4. As expected, the maximum dry cell weight (DCW) in the tungsten-added medium was greater than that in the tungsten-free medium (2.29 g/L vs 0.69 g/L at 96 h) (Fig. 4A), and the maximum production of ectoine remarkably increased up to 2.3-fold in the tungsten-added medium compared with the tungsten-free medium (31.43 mg/L at 48 h vs. 13.85 mg/L at 72 h) (Fig. 4B). Ectoine yield (mg/DCW g) was also increased up to 1.38-fold in the tungsten-added medium until 48 h before ectoine production rapidly decreased in the tungsten-added medium. These results suggest that tungsten addition plays a positive role in both biomass production and ectoine production in *M. alcaliphilum* 20ZDP. Thus, tungsten (0.05 μM) was added to the medium in all subsequent batch cultures. Interestingly, ectoine accumulated slowly until 48 h and was maintained or decreased slightly in the tungsten-free medium, whereas ectoine production increased rapidly until 48 h and then decreased remarkably in the tungsten-added medium. It is assumed that this is because the ectoine might be reused as a cell component for cell growth in tungsten-added medium.

### Construction of the *ectD* deletion mutant and batch cultivation of the mutant using methane

**Fig. 5:**
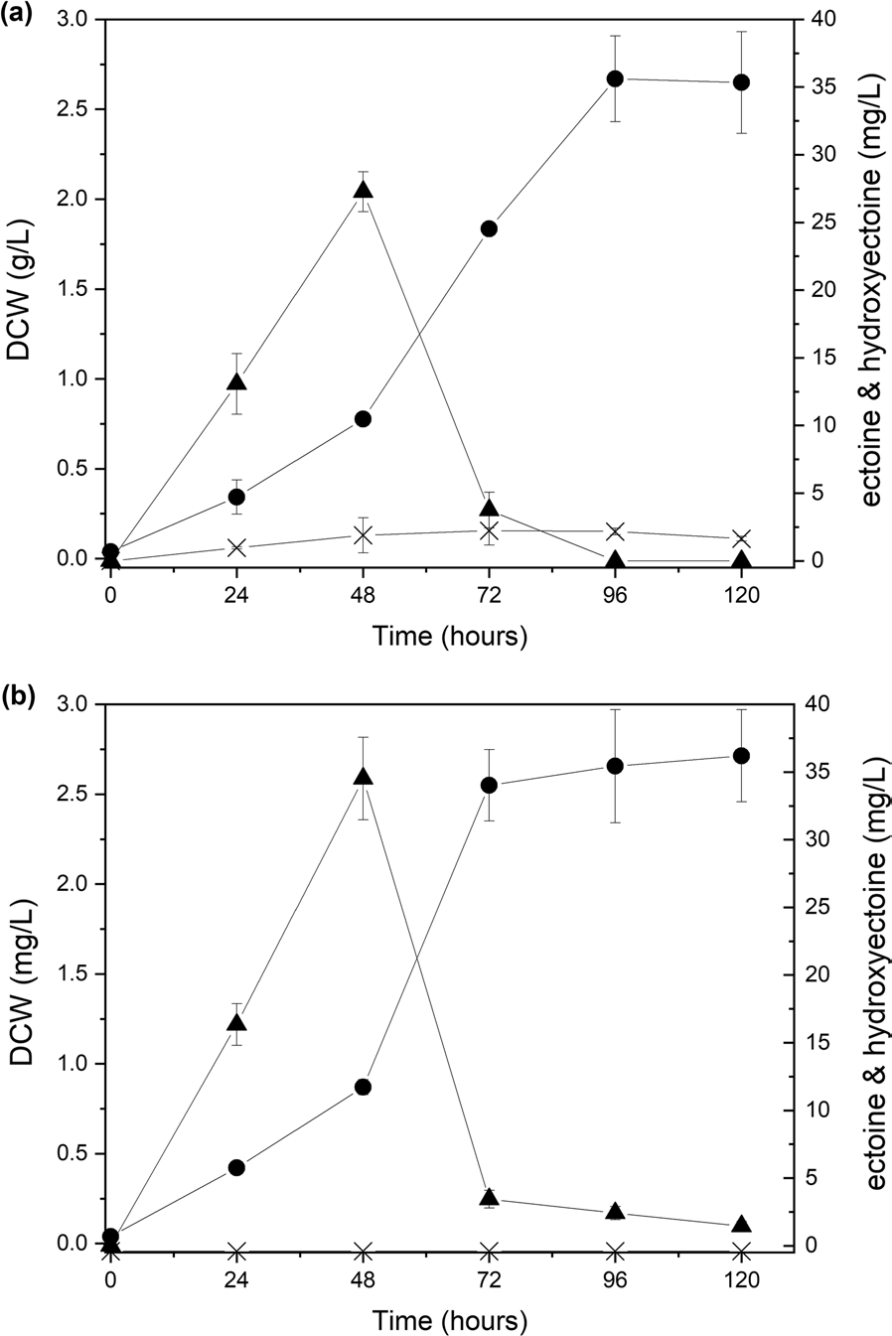
Time course of the metabolite profile and growth of *M. alcaliphilum* 20ZDP (**a**) and *M. alcaliphilum* 20ZDP1 (**b**). The following symbols were used: DCW (●), ectoine (▲), and hydroxyectoine (X). All experiments were performed in triplicate and the range of the raw data was within ± 5% of the average

Although *M. alcaliphilum* 20ZDP could produce ectoine using methane as a carbon source, the formation of the by-products needed to be decreased for efficient ectoine production. It was confirmed that the production ratio of hydroxyectoine to ectoine accounts for approximately 4–8% by *M. alcaliphilum* 20ZDP cultivated in *Methylomicrobium* medium containing 3% NaCl. To reduce the formation of hydroxyectoine, the *ectD* gene encoding ectoine hydroxylase, which is responsible for the synthesis of hydroxyectoine from ectoine, was deleted in the chromosome using the sucrose counter-selection method. PCR and nucleotide sequencing data (data not shown) confirmed that the *ectD* gene of *M. alcaliphilum* 20ZDP was successfully deleted (Additional file 1: Fig. S2), and this mutant strain was named *M. alcaliphilum* 20ZDP1 (Table 1). When batch flask fermentation was conducted with the *M. alcaliphilum* 20ZDP1 strain in *Methylomicrobium* medium containing 3% NaCl, *ectD* deletion had a positive effect on ectoine production compared to *M. alcaliphilum* 20ZDP without the inhibition of cell growth (Fig. 5). Furthermore, the deletion of the *ectD* gene in *M. alcaliphilum* 20ZDP1 resulted in abolished hydroxyectoine formation, compared with that observed in *M. alcaliphilum* 20ZDP (2.2 mg/L at 72 h). *Methylomicrobium alcaliphilum* 20ZDP1 showed more ectoine production than *M. alcaliphilum* 20ZDP (34.5 mg/L vs. 27.3 mg/L). This demonstrates that the disruption of *ectD* not only completely inhibited hydroxyectoine production, but also induced more ectoine production.

**Table 1:** Strains and plasmids used in this study

	Characteristics	References or source
Strains		
* Escherichia coli* DH10b		
* Escherichia coli* S17-1 λ*pir*	Donor strain	
* Methylomicrobium alcaliphilum* 20Z	Used as host strain	DSMZ
* M. alcaliphilum* 20ZDP	Mutant of *M. alcaliphilum* 20Z which was removed an endogenous plasmid by knocking out *repB* in endogenous plasmid locus	This study
* M. alcaliphilum* 20Z/dt	*M. alcaliphilum* 20Z harboring pAWP89	This study
* M. alcaliphilum* 20ZDP/dt	*M. alcaliphilum* 20ZDP harboring pAWP89	This study
* M. alcaliphilum* 20ZDP1	Mutant of *M. alcaliphilum* 20ZDP which was deleted an *ectD*	This study
* M. alcaliphilum* 20ZDP2	Mutant of *M. alcaliphilum* 20ZDP which was deleted an *ectD* and *ectR*	This study
Vectors		
pAWP89	IncP-based broad host-range plasmid	
pK19mobsacB	Kanamycin resistant version of suicide vector pK19mobsacB	This study
pK19△*repB*	pK19mobsacB containing flank regions of *repB*	This study
pK19△*ectD*	pK19mobsacB containing flank regions of *ectD*	This study
pK19△*ectD*△*ectR*	pK19mobsacB delete containing flank regions of *ectR*	This study
*M. alcaliphilum* 20ZDP2	Mutant of *M. alcaliphilum* 20ZDP which was deleted an *ectD* and *ectR*	This study

In previous study, deletion of genes encoding ectoine hydroxylase (*EctD*) and ectoine hydrolase (*DoeA*) that responsible for ectoine catabolism were resulted in an obviously enhanced productivity of ectoine in *H. hydrothermalis* Y2 [32]. A significant increase in ectoine production was observed by simultaneous deletion of *ectD* and *doeA* rather than deletion of *ectD* alone in *H. hydrothermalis* Y2. Since it was also found that ectoine was degraded in *M. alcaliphilum* 20Z, it is expected that ectoine production can be increased by deletion of gene that responsible for ectoine catabolism in further study.

### Construction of *ectD* and *ectR* double deletion mutant and batch cultivation of the double mutant using methane

The genes encoding key enzymes of the ectoine synthesis pathway in *M. alcaliphilum* 20Z were organized in the *ectABC* operon. Upstream of this gene cluster, the *ectR* gene encoding a MarR-like transcription regulator (EctR) was identified in *M. alcaliphilum* 20Z, and was indicated to be a negative regulator of the *ectABC* operon [27]. To investigate the effect of EctR on ectoine biosynthesis in *M. alcaliphilum* 20Z, we created the double deletion mutant strain *M. alcaliphilum* 20ZDP Δ*ectD*Δ*ectR* which was named *M. alcaliphilum* 20ZDP2 (Table 1). This was done by removing the *ectR* gene from the Δ*ectD* mutant (*M. alcaliphilum* 20ZDP1). Successful deletion of *ectR* was confirmed by PCR amplification (Additional file 1: Fig. S2) and nucleotide sequencing data (data not shown).

**Fig. 6:**
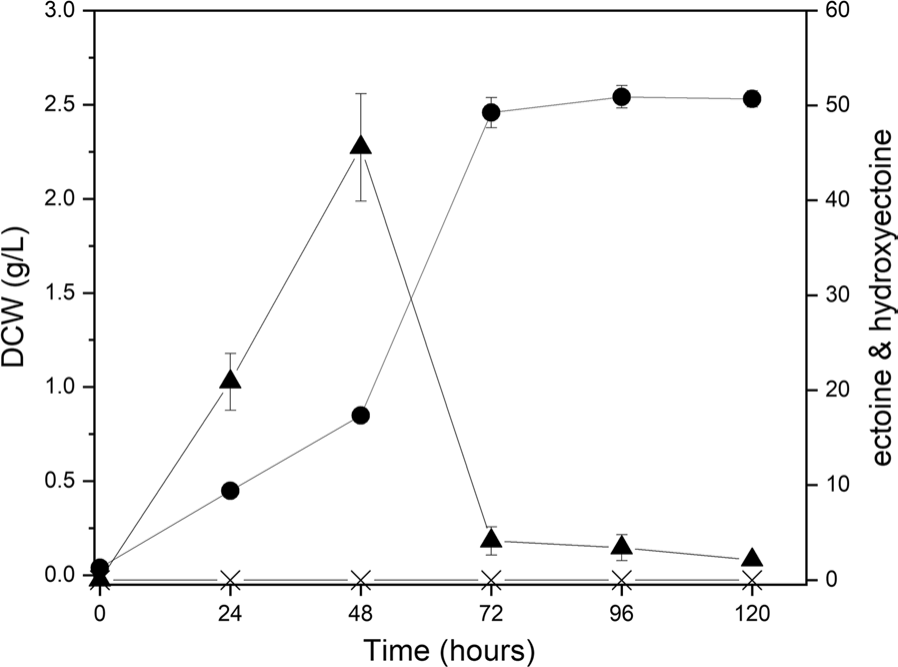
Time course of *M. alcaliphilum* 20ZDP2 metabolite profile and growth with methane as carbon source. Following symbols were used: DCW (●), ectoine (▲), and hydroxyectoine (X). All experiments were performed in triplicate and the range of the raw data was within ± 5% of the average

To confirm the effect of the deletion of *ectD* and *ectR* on ectoine production, batch cultures in flasks were conducted with methane as the carbon source using *M. alcaliphilum* 20ZDP2 (Fig. 6). Batch fermentation was performed for 96 h, and cell growth and intracellular ectoine and hydroxyectoine were measured every 24 h. The maximum ectoine concentration reached 45.58 mg/L at 48 h without hydroxyectoine production, and then rapidly decreased. As shown in Figs. 5 and 6, ectoine production increased up to 1.3-fold compared to that of *M. alcaliphilum* 20ZDP1 (45.58 mg/L vs. 34.53 mg/L)*.* These results clearly indicate that the deletion of *ectD* and *ectR* had a beneficial effect on ectoine production without the inhibition of cell growth. More importantly, the disruption of *ectD* resulted in the inhibition of hydroxyectoine production, and the deletion of *ectD* and *ectR* further induced ectoine production, resulting in a remarkable increase in ectoine yield, which was up to 1.6-fold of that obtained with *M. alcaliphilum* 20ZDP (45.58 mg/L vs. 27.26 mg/L).

### Enhanced production of ectoine from methane by *M. alcaliphilum* 20ZDP2 in batch cultivation

To improve ectoine production by *M. alcaliphilum* 20ZDP2, some process parameters such as agitation speed and temperature were examined. When the agitation speed was adjusted to 150, 230, and 300 rpm, there was no significant change in the cell growth and ectoine production by *M. alcaliphilum* 20ZDP2 (Additional file 1: Fig. S3). However, when the temperature was adjusted to 25 °C, 30 °C, and 35 °C, the cell growth and ectoine production were the best at 30 °C (Additional file 1: Fig. S4) by using *M. alcaliphilum* 20ZDP2. Therefore process parameters for ectoine production were determined to be 230 rpm and 30 °C.

**Fig. 7:**
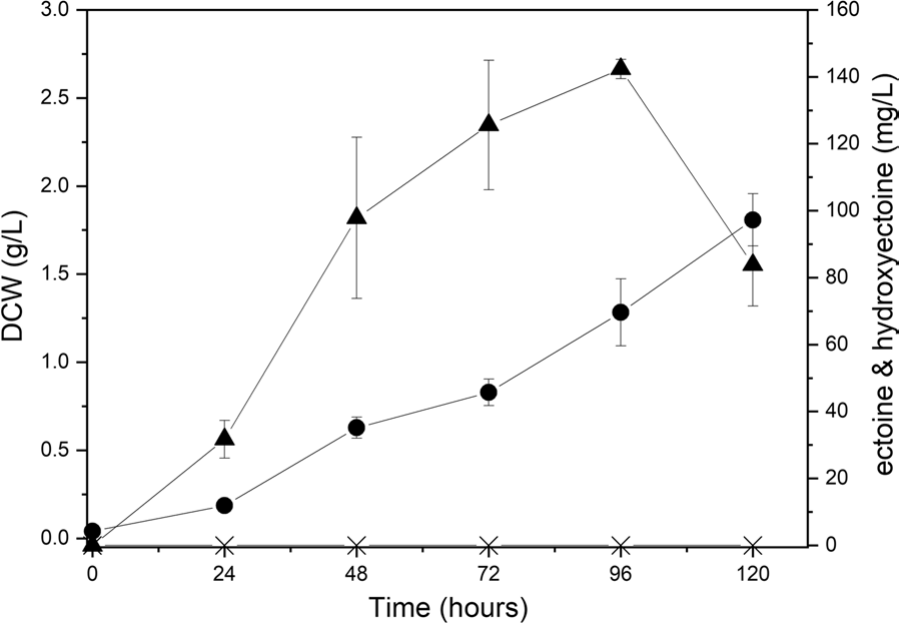
High production of ectoine from methane by *M. alcaliphilum* 20ZDP2 in optimized medium. Growth medium was *Methylomicrobium* medium containing 6% NaCl and 0.05 μM tungsten. Following symbols were used: DCW (●), ectoine (▲), and hydroxyectoine (X). All experiments were performed in triplicate and the range of the raw data was within ± 5% of the average

NaCl concentration is a major factor in the production of intracellular ectoine in *M. alcaliphilum* 20Z [24]. To examine the salinity adaptation range and the optimal concentration of NaCl for intracellular ectoine production in *M. alcaliphilum* 20ZDP2, media of varying salinity (3%, 6%, and 9% NaCl) were used and cell growth and intracellular ectoine production were comparatively evaluated. It was observed that the growth was severely inhibited in a medium containing 9% NaCl (data not shown). As shown in Figs. 6 and 7, it was confirmed that the higher the salinity of the medium, the lower the cell growth rate. However, when *M. alcaliphilum* 20ZDP2 was cultivated in a medium containing 6% NaCl, DCW increased up to 120 h and finally reached 1.81 g/L. Meanwhile, maximum ectoine production was 3.1-fold higher (142.32 mg/L at 96 h) than that obtained with 3% NaCl-containing medium (45.58 mg/L at 48 h) (Figs. 6 and 7). No hydroxyectoine was detected in any of the experiments using *M. alcaliphilum* 20ZDP2. Moreover, the maximum ectoine yield (mg/DCW g) was 2.1-fold higher in the medium containing 6% NaCl than in the medium containing 3% NaCl. As seen in Figs. 4 and 7, 0.05 μM tungsten- and 6% NaCl-containing medium was the most effective for ectoine production by *M. alcaliphilum* 20ZDP2. Comparing Figs. 6 and 7, it can be observed that ectoine production dramatically increased with increasing salinity, even when the *ectR* gene was deleted. These results demonstrated that the *ectABC-ask* operon was activated in response to the high osmolarity of the growth medium, but ectR1 is most likely not essential for the activation of the *ectABC* operon in response to elevated salinity. Thus, EctR-mediated control of the *ectABC-ask* operon is not a single mechanism, and an alternative uncharacterized regulatory system of ectoine production might exist in *M. alcaliphilum* 20Z.

**Fig. 8:**
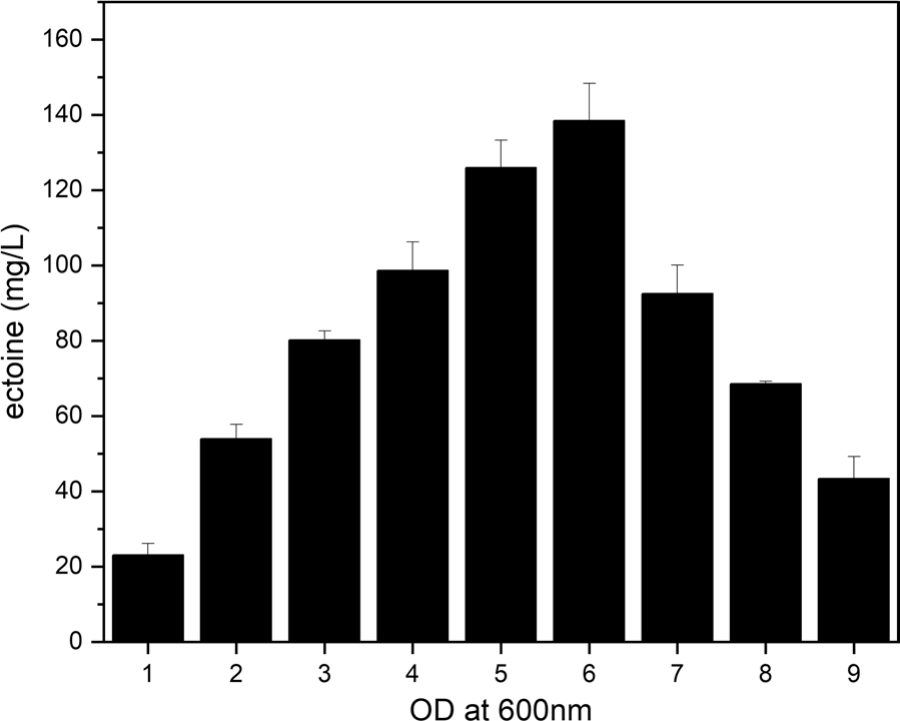
Comparison of ectoine production according to cell growth. *Methylomicrobium alcaliphilum* 20ZDP2 was cultured in medium containing 6% NaCl and 0.05 μM tungsten and sampled each time as the OD_600_ value increased (OD 1–9). All experiments were performed in triplicate and the range of the raw data was within ± 5% of the average

The correlation between ectoine production and the growth phase in *M. alcaliphilum* 20Z has been previously reported in batch cultivation [33]. Intracellular ectoine production was the highest in the mid-exponential growth phase of *M. alcaliphilum* 20Z, and decreased rapidly during the stationary growth phase, suggesting that accumulated ectoine might be used for the synthesis of cell constituents [24, 34]. The fact that extracellular ectoine was not detected at all when intracellular ectoine rapidly decreased indicates that ectoine was not excreted outside of cells at that point but was degraded inside. Therefore, maximum ectoine production can be achieved by identifying the appropriate time to stop the culture before the rapid degradation of ectoine. In order to determine an appropriate growth phase for the maximum production of ectoine, *M. alcaliphilum* 20ZDP2 was cultured in a medium containing 0.05 μM tungsten and 6% NaCl, along with methane as the sole carbon source, and sampled each time the OD_600_ value increased (OD 1–9) while the accumulated ectoine was compared according to the growth phase. As shown in Fig. 8, the accumulation of ectoine increased with cell growth, and when the cell optical density was 6, the cells could synthesize the most ectoine up to 138.44 ± 9.93 mg/L. After reaching the highest ectoine synthesis, it decreased dramatically with cell growth.

Reshetnikov et al. [35] showed that *M. alcaliphilum* 20Z possesses the *doeBDAC* gene cluster, which is responsible for ectoine degradation. This gene cluster codes for putative ectoine hydrolase (DoeA), Nα-acetyl-l-2,4-diaminobutyrate deacetylase (DoeB), diaminobutyrate transaminase (DoeD), and aspartate semialdehyde dehydrogenase (DoeC). These four enzymes catalyze ectoine hydrolysis, producing *N*-acetyl-DAB, and further deacetylate it to diaminobutyrate (DAB) and acetate (Fig. 1) [36]. DAB can either flow off to aspartate or re-enter the ectoine synthesis pathway. Therefore, it is expected that the genetic manipulation of genes involved in ectoine degradation can further increase ectoine production by preventing the rapid degradation of the accumulated ectoine.

Table 2 compares ectoine production from glucose and methane from previous reports and this study. The main process for ectoine production was focused on sugar fermentation using *Halomonas* species and metabolically engineered *E. coli* and *C. glutamicum*. Until now, Gießelmann et al. [22] reported the highest ectoine production (65.3 g/L) from glucose and molasses by metabolically engineered *C. glutamicum.* Zhao et al. [32] obtained 10.5 g/L of ectoine with the yield of 0.22 g/DCW g by *H. hydrothermalis* Y2 from glucose and sodium glutamate. However, this process is costly due to the need for high quality carbon source such as glucose, yeast, and sodium glutamate [37]. *M. alcaliphilum* 20Z was able to accumulate ectoine up to 70 mg/g DCW during the treatment of dilute methane emission under continuous mode [10]. Additionally, a recent study demonstrated that *M. alcaliphilum* 20Z and a mixed haloalkaliphilic consortium were able to produce ectoine up to 109 mg/g DCW using upgraded biogas under continuous mode [38]. So far, previous studies on ectoine production from methane have been conducted using parental strain due to limitations in genetic manipulation of methanotrophs, and it was focused on bio-milking process to enhance ectoine production. In this study, we constructed *M. alcaliphilum* 20ZDP with a high conjugation efficiency and stability of episomal plasmid by removal of native plasmid to facilitate genetic manipulation of the strain. And then, the enhanced ectoine production from methane was fulfilled by deletion of *ectD* and *ectR* in *M. alcaliphilum* 20ZDP in a medium containing 6% of NaCl and 0.05 μM of tungsten. Overall, a new record of the highest ectoine concentration from methane (142.32 mg/L) as the sole carbon source was achieved without hydroxyectoine production.

**Table 2:** Comparison of ectoine production

Host bacteria	Carbon sources	NaCl (M)	Culture mode	Ectoine titer (g/L)	Ectoine yield (g/DCW g)	References
*Halomonas* *Elongate* DSM142	Glucose	Bacterial milking	Fed-batch	7.4	0.2	[33]
*Halomonas* *hydrothermalis* Y2	Monosodium glutamate, glucose	1	Fed-batch	10.5	0.22	[32]
*Escherichia* *coli*	Glucose	–	Fed-batch	30.4	–	[21]
*Corynebacterium glutamicum ectABC*^*opt*^	Glucose, molasses	0.03	Fed-batch	65.3	–	[22]
*Methylomicrobium alcaliphilum* 20Z	Methane	1	Continuous mode	0.047	0.070	[10]
*Methylomicrobium alcaliphilum* 20Z, mixed haloalkaliphilic consortium	Biogas (CH_4_, O_2_, CO_2_, He)	1	Continuous mode	–	0.109	[38]
*Methylomicrobium alcaliphilum* 20ZDP2	Methane	1	Flask	0.143	0.111	This study

## Conclusions

In this study, we constructed *M. alcaliphilum* 20ZDP with a high conjugation efficiency and stability of episomal plasmids by the removal of its native plasmid. Enhanced ectoine production from methane was achieved by disrupting the *ectD* and *ectR* genes, which resulted in the inhibition of hydroxyectoine production. Further improvement of ectoine production (yield up to 142.32 mg/L) without the production of hydroxyectoine was achieved under optimized conditions using a medium containing 6% NaCl and 0.05 μM tungsten. In particular, in contrast with the results of previous studies using the *M. alcaliphilum* 20Z parental strain, the results of this study showed that the deletion of *ectD* and *ectR* was effective in the enhancement of ectoine production. To the best of our knowledge, the ectoine concentration achieved in this study is a new record for ectoine production with the *M. alcaliphilum* 20Z strain.

## Methods

### Microorganisms and medium

The strain used in this study, *Methylomicrobium alcaliphilum* 20Z (equivalent to DSMZ19304) was purchased from DSMZ. *Methylomicrobium alcaliphilum* 20Z was cultivated in a 250-mL baffled flask sealed with a screw cap, containing 50 mL of *Methylomicrobium* medium consisting of (per L of distilled water) NaCl, 30.0 g; MgSO_4_ 7H_2_O, 0.2 g; CaCl_2_ 2H_2_O, 0.02 g; KNO_3,_ 1.0 g; and trace elements, 1 mL; supplemented with 20 mL phosphate buffer (KH_2_PO_4,_ 14.0 g/L; Na_2_HPO_4_ · 12H_2_O, 30 g/L), 50 mL of 1 M NaHCO_3_, and 5 mL of 1 M Na_2_CO_3_. In flask fermentation, methane and air mixtures (3:7) were directly purged into the head space of the baffled flask and refreshed every 12 h. To create a high osmotic growth environment, NaCl was added to the medium as needed (30–90 g/L). To select the strains containing the recombinant plasmids, kanamycin was added at a final concentration of 50 μg/mL.

### Cultivation

Culturing was performed in a baffled flask at 30 °C, shaken at 230 rpm. For methane fermentation, the headspace:medium ratio was 4:1. The methane and air composition in the headspace was determined using a mass flow controller (Alicat Mass Flow Controller, Alicat Scientific Inc., AZ, USA). The seed culture was inoculated into 50 mL of *Methylomicrobium* medium supplemented with methanol (1%, v/v) and cultivated for 2 days at 30 °C, shaken at 230 rpm and transferred to a 250-mL baffled flask containing 50 mL of *Methylomicrobium* medium for the main culture. The main culture was inoculated (final OD_600_ at 0.2) into a 250-mL baffled flask containing 50 mL of the *Methylomicrobium* medium, supplied with methane as a carbon source; the medium was refreshed every 12 h. Tungsten (0.05 μM) was added to the *Methylomicrobium* medium as needed. Cells were collected every 24 h for the analysis of the optical density and presence of any metabolites (ectoine and hydroxyectoine). All experiments were performed in triplicate.

### Genetic manipulation of *M. alcaliphilum* 20Z

All genetic manipulations of *M. alcaliphilum* 20Z were performed using the conjugation method described in a previous study [28]. Briefly, 50 mL each of *M. alcaliphilum* 20Z recipient culture and *Escherichia coli* S17-1 λpir donor culture were grown to optical densities (at 600 nm) of 0.4 and 0.6, respectively. Cells were harvested after centrifugation at 4000 rpm at 4 °C for 10 min. The collected cells were mixed and spread on a mating plate containing 2 g/L NaCl and 15% nutrient broth in the DSMZ medium. The mixture was incubated at 30 °C for 2 days and then spread on a selection plate containing 50 μg/mL kanamycin and 10 μg/mL nalidixic acid in the *Methylomicrobium* medium. Deletion mutagenesis was performed as described previously [29]. The flanking region of each gene was amplified by PCR from the chromosome of *M. alcaliphilum* 20Z and cloned into the suicide vector pK19mobsacB. For the sucrose counter-selection, single-crossover kanamycin-resistant *M. alcaliphilum* 20Z colonies were spread on a selection medium containing 5% sucrose to generate double-crossover colonies. Deletion of the genes from the chromosome was confirmed by the size of the PCR products obtained using the oligonucleotide pair. The plasmids and strains used in this study are listed in Table 1. All oligonucleotides were synthesized by Bionics Co. (Seoul, Korea) and are listed in Table 3. For the molecular plasmid cloning, the In-Fusion HD cloning kit and all enzymes used in this study were purchased from Takara Bio (Shiga, Japan).

**Table 3:** Oligonucleotides used in this study

Oligonucleotides	Sequence
repBDown F	TGACATGATTACGCCAAGCTTATGAAGGAATCGTTGTCGTACCC
repBDown R	ATATCGCCGCATTGGCAGGTTACGACCCTAAGC
repBUp F	ACCTGCCAATGCGGCGATATGGGGAAAAT
repBUp R	AAAACGACGGCCAGTGAATTCTTATGAAATGATCCGCCGTTTGTGC
repBDI F	TGTGTGGAATTGTGAGCGGA
repBDI R	GGTCGAGTGGCTTAAAACGC
repBUI F	GCCTTTCAACGGCCATCTTC
repBUI R	TTTCTGCGGACTGGCTTTCT
repB confirm F	TCCTTGGCCACCGAATTACC
repB confirm R	GCACTTTGCAACCCGACAAT
korB confirm F	ACCGTCTTGAGTTGGTCGTC
korB confirm R	AACTAAGAGCTTCGGCCCAC
trbF confirm F	CTAACCCGTACCTGACTGCC
trbF confirm R	ACGCACATAGATACCGAGCG
ectDDown F	TGACATGATTACGCCAAGCTTTGTTCAGCAACAAAAGACAGAA
ectDDown R	ATCACGTAACATGCTTAGAATGTTAATAATGTTGA
ectDUp F	TTCTAAGCATGTTACGTGATTGATAAAAATCTTCA
ectDUp R	AAAACGACGGCCAGTGAAAGTGAATTCTTGTACTGATGTTTCTTACCCCT
ectDDI F	CACTCATTAGGCACCCCAGG
ectDDI R	CAATGGAAAACCCGGCAGTG
ectDUI F	CACTGCCGGGTTTTCCATTG
ectDUI R	ACGACGGCCAGTGAATTCTT
ectD confirm F	TGCTTCATCGTTGACGCTCT
ectD confirm R	ATGGCAAGTCAGTCGAGCAA
ectR Down F	TGACATGATTACGCCAAGCTTTCTAATTCTCTCCTGAGCAAGATG
ectR Down R	TTATTAGAGCCATTGATCACCAATCATTAGAATAG
ectRUp F	GTGATCAATGGCTCTAATAATTCAGCTCAGCT
ectRUp R	AAAACGACGGCCAGTGAATTCAGAACATCAAGAGGTCTGGATTGT
ectRDI F	TTTGCTGGCCTTTTGCTCAC
ectRDI R	GTAAATCGGTGGCGCGAATC
ectRUI F	TACGTAGAGTGATTCGCGCC
ectRUI R	CGGACTGGCTTTCTACGTGT
ectR confirm F	GGGCATTTGCTAATCAGCCG
ectR confirm R	AACAGCACCGCTTCCAAGTA

### dTomato reporter assay

Here, 20 mL of liquid culture was harvested by centrifugation at 4000 rpm at 4 °C for 10 min. Cell pellets were resuspended in 1 mL of 50 mM Tris buffer (pH 7.5) and sonicated using a Sonicator (Sonosmasher, Yeonjin Co., Seoul, Korea). After centrifuging at 14,000 rpm at 4 °C for 30 min, 100 μL of the supernatant was assayed in a 96-well plate to measure fluorescence using a microplate fluorometer (Fluorokan Ascent FL, Thermo Fisher Scientific, MA, USA) with excitation/emission of 544/590 nm. The total protein concentration was determined by Bradford protein assay using Bradford Reagent (Quick Start™ Bradford 1X Dye Reagent, Bio-Rad, CA, USA); the optical density was measured at 595 nm.

### Analytical methods

Dry cell weight (DCW, g/L) was calculated based on the optical density at 600 nm (OD_600_) using a calibration curve of OD_600_ vs. dry cell weight of *M. alcaliphilum* 20Z as described in a previous study [31]. The observed DCW parameters were as follows: 1 L of cell culture with an OD_600_ of 1 corresponded to 0.198 ± 0.031 g DCW.

The OD_600_ of the broth was measured using a UV spectrophotometer (Biochrom WPA Lightwave II, Biochrom Ltd., Cambridge, UK) with appropriate dilution.

Ectoine extraction was performed according to a previously reported method [30]. To determine the intracellular ectoine, harvested cells were freeze-dried for 48 h (TFD8503, Ilshin BioBase Co., Ltd., Gyeonggi, Korea), and 10 mg of cell mass was extracted using 570 μL of ectoine extraction solution (methanol/chloroform/water, 10:5:4, v/v) by vigorous shaking for 5 min followed by the addition of 170 μL of chloroform and water. The mixture was shaken again for 10 min, and the phase separation was enhanced by centrifugation. The hydrophilic top layer was collected and diluted and the ectoine concentration was measured by HPLC (DGU-20A degassing unit, LC-20AD pump, SIL-20A automatic injector, RID-20A refractive index, SPD-20A UV–Vis detector, and CTO-20A column oven, Shimadzu, Kyoto, Japan) equipped with a refractive index detector and Zorbox-NH2 Column (Analytical, 4.6 × 250 mm, 5 μm) under the following conditions: sample volume, 10 μL; mobile phase, 70% acetonitrile (v/v); flow rate, 1 mL/min; and column temperature, 40 °C [19]. To measure the extracellular ectoine, the culture broth was centrifuged and the supernatant was mixed with acetonitrile in a 1:1 ratio and analyzed using HPLC as described above.

## Supplementary Information

**Additional file 1: Figure S1.** PCR verification for the removal of the native plasmid in *M. alcaliphilum* 20Z. The electrophoresis bands by colony PCR at three different loci (*repB*, *korB*, and *trbF*) on the native plasmid. Lanes 1, 2, and 3, PCR products of *M. alcaliphilum* 20Z with *repB* confirm, *KorB* confirm, and *trbF* confirm primer pairs; Lane 4, molecular weight marker; Lane 5, 6, and 7, PCR products of *M. alcaliphilum* 20ZDP with *repB* confirm, *KorB* confirm, and *trbF* confirm primer pairs. **Figure S2.** PCR verification for the removal of the *ectD* and e*ctR* genes in *M. alcaliphilum* 20Z. (a) The electrophoresis bands observed after the colony PCR of e*ctD* in *M. alcaliphilum* 20ZDP and *M. alcaliphilum* 20ZDP1. Lane 1, PCR product of *M. alcaliphilum* 20ZDP with *ectD* confirm primer pair; Lane 2, molecular weight marker; Lane 3, PCR product of *M. alcaliphilum* 20ZDP1 with *ectD* confirm primer pair (b) The electrophoresis bands observed after the colony PCR of *ectD* and *ectR* in *M. alcaliphilum* 20ZDP and *M. alcaliphilum* 20ZDP2. Lane 1, PCR product of *M. alcaliphilum* 20ZDP with *ectD* confirm primer pair; Lane 2, PCR product of *M. alcaliphilum* 20ZDP with *ectR* confirm primer pair; Lane 3, molecular weight marker; Lane 4, PCR product of *M. alcaliphilum* 20ZDP2 with *ectD* confirm primer pair; Lane 5, PCR product of *M. alcaliphilum* 20ZDP2 with *ectR* confirm primer pair. **Figure S3.** Effect of agitation speed on cell growth and ectoine production in *M. alcaliphilum* 20ZDP2. *M. alcaliphilum* 20ZDP2 was cultivated in a *Methylomicrobium* medium containing 6% NaCl and 0.05 μM of tungsten at 30 °C shaken at different agitation speed. (a) dry cell weight (DCW) and (b) ectoine production. The following symbols were used: 150 rpm (■), 230 rpm (●), and 300 rpm (▲). All experiments were performed in triplicate and the range of the raw data was within ± 5% of the average. **Figure S4.** Effect of temperature on cell growth and ectoine production in *M. alcaliphilum* 20ZDP2. *M. alcaliphilum* 20ZDP2 was cultivated in a *Methylomicrobium* medium containing 6% NaCl and 0.05 μM of tungsten shaken 230 rpm at different temperature. (a) dry cell weight (DCW) and (b) ectoine production. The following symbols were used: 25 °C (■), 30 °C (●), and 35 °C (▲). All experiments were performed in triplicate and the range of the raw data was within ± 5% of the average.

## Data Availability

All data generated during this study are included in this article and the additional files. Raw data are available on reasonable request.

## Abbreviations

Ask: Aspartate kinase
AsdH: B-aspartate-semialdehyde-dehydrogenase
EctB: l-2,4-Diaminobutyrate transaminase
EctA: l-2,4-Diaminobutyrate*N*γ-acetyltransferase
EctC: Ectoine synthase
EctD: Ectoine hydroxylase
DoeA: Ectoine hydrolase
DoeB: *N*α-Acetyl-l-2,4-diaminobutyrate deacetylase
DoeD: l-2,4-Diaminobutyrate transaminase
DoeC: Aspartate-semialdehyde dehydrogenase
DAB: Diaminobutyrate
DCW: Dry cell weight
OD_600_: Optical density at 600 nm
PCR: Polymerase chain reaction
