# Supplementary material for: Enhanced production of ectoine from methane using metabolically engineered Methylomicrobium alcaliphilum 20Z
Source: Biotechnol Biofuels Bioprod. 2022 Jan 13;15:5. doi: 10.1186/s13068-022-02104-2 (PMC8759281; doi:10.1186/s13068-022-02104-2)
Supplement: Supplementary file 1 — Additional file 1: Figure S1. PCR verification for the removal of the native plasmid in M. alcaliphilum 20Z. The electrophoresis bands by colony PCR at three different loci (repB, korB, and trbF) on the native plasmid. Lanes 1, 2, and 3, PCR products of M. alcaliphilum 20Z with repB confirm, KorB confirm, and trbF confirm primer pairs; Lane 4, molecular weight marker; Lane 5, 6, and 7, PCR products of M. alcaliphilum 20ZDP with repB confirm, KorB confirm, and trbF confirm primer pairs. Figure S2. PCR verification for the removal of the ectD and ectR genes in M. alcaliphilum 20Z. (a) The electrophoresis bands observed after the colony PCR of ectD in M. alcaliphilum 20ZDP and M. alcaliphilum 20ZDP1. Lane 1, PCR product of M. alcaliphilum 20ZDP with ectD confirm primer pair; Lane 2, molecular weight marker; Lane 3, PCR product of M. alcaliphilum 20ZDP1 with ectD confirm primer pair (b) The electrophoresis bands observed after the colony PCR of ectD and ectR in M. alcaliphilum 20ZDP and M. alcaliphilum 20ZDP2. Lane 1, PCR product of M. alcaliphilum 20ZDP with ectD confirm primer pair; Lane 2, PCR product of M. alcaliphilum 20ZDP with ectR confirm primer pair; Lane 3, molecular weight marker; Lane 4, PCR product of M. alcaliphilum 20ZDP2 with ectD confirm primer pair; Lane 5, PCR product of M. alcaliphilum 20ZDP2 with ectR confirm primer pair. Figure S3. Effect of agitation speed on cell growth and ectoine production in M. alcaliphilum 20ZDP2. M. alcaliphilum 20ZDP2 was cultivated in a Methylomicrobium medium containing 6% NaCl and 0.05 μM of tungsten at 30 °C shaken at different agitation speed. (a) dry cell weight (DCW) and (b) ectoine production. The following symbols were used: 150 rpm (■), 230 rpm (●), and 300 rpm (▲). All experiments were performed in triplicate and the range of the raw data was within ± 5% of the average. Figure S4. Effect of temperature on cell growth and ectoine production in M. alcaliphilum 20ZDP2. M. alcaliphilum 20ZDP2 was cultivated in a Met [file 13068_2022_2104_MOESM1_ESM.pdf]

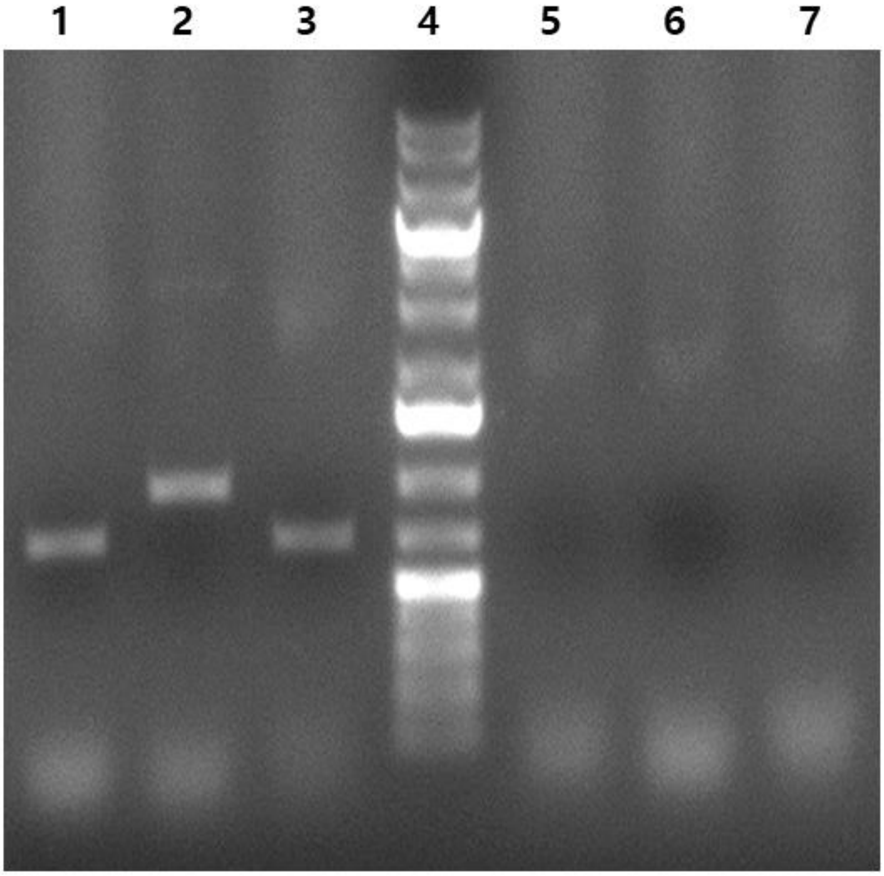

Figure S1. PCR verification for the removal of the native plasmid in *M. alcaliphilum* 20Z. The electrophoresis bands by colony PCR at three different loci (*repB*, *korB*, and *trbF*) on the native plasmid. Lanes 1, 2, and 3, PCR products of *M. alcaliphilum* 20Z with *repB* confirm, *KorB* confirm, and *trbF* confirm primer pairs; Lane 4, molecular weight marker; Lane 5, 6, and 7, PCR products of *M. alcaliphilum* 20ZDP with *repB* confirm, *KorB* confirm, and *trbF* confirm primer pairs.

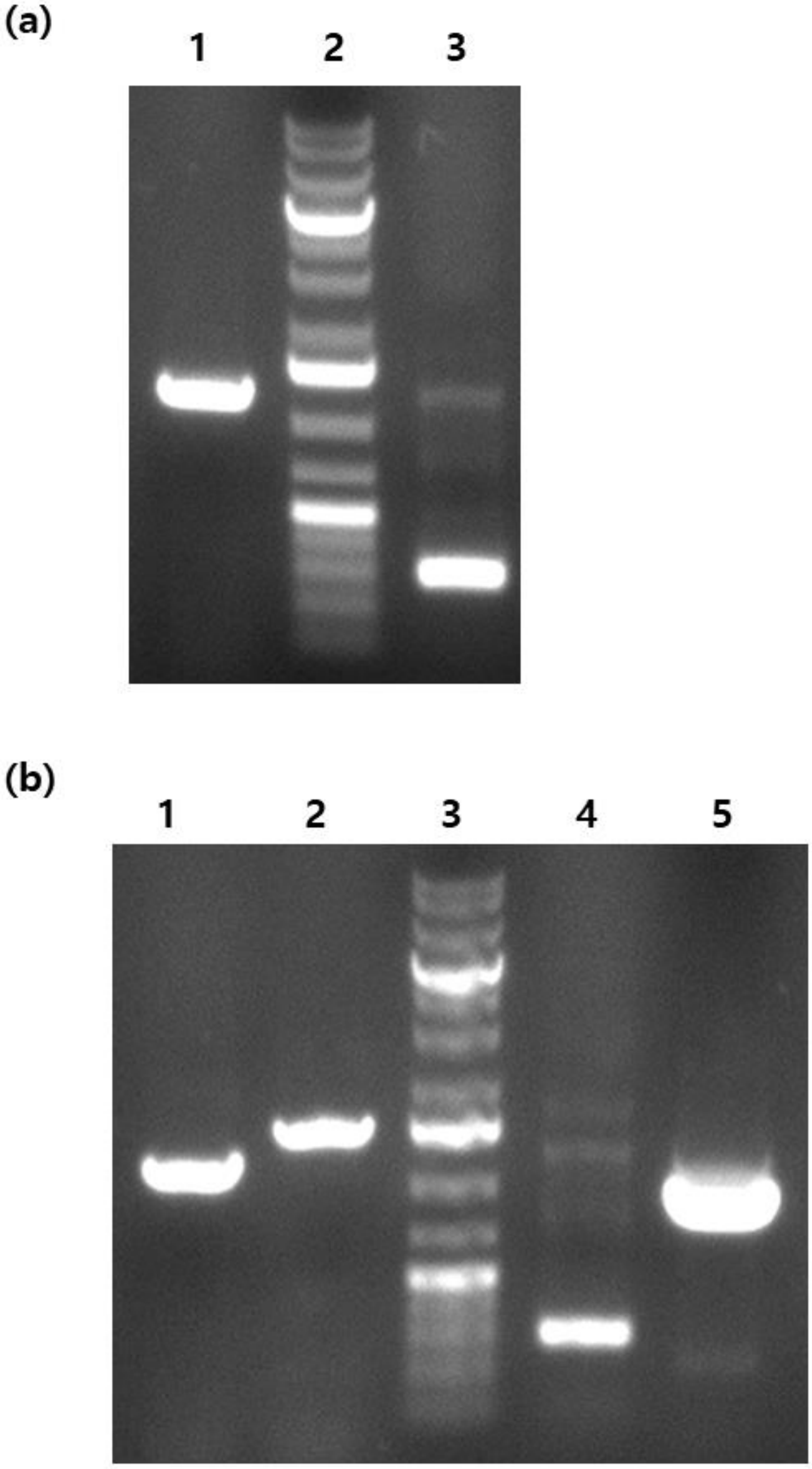

Figure S2. PCR verification for the removal of the *ectD* and e*ctR* genes in *M. alcaliphilum* 20Z. (a) The electrophoresis bands observed after the colony PCR of e*ctD* in *M. alcaliphilum*

20ZDP and *M. alcaliphilum* 20ZDP1. Lane 1, PCR product of *M. alcaliphilum* 20ZDP with *ectD* confirm primer pair; Lane 2, molecular weight marker; Lane 3, PCR product of *M. alcaliphilum* 20ZDP1 with *ectD* confirm primer pair (b) The electrophoresis bands observed after the colony PCR of *ectD* and *ectR* in *M. alcaliphilum* 20ZDP and *M. alcaliphilum* 20ZDP2. Lane 1, PCR product of *M. alcaliphilum* 20ZDP with *ectD* confirm primer pair; Lane 2, PCR product of *M. alcaliphilum* 20ZDP with *ectR* confirm primer pair; Lane 3, molecular weight marker; Lane 4, PCR product of *M. alcaliphilum* 20ZDP2 with *ectD* confirm primer pair; Lane 5, PCR product of *M. alcaliphilum* 20ZDP2 with *ectR* confirm primer pair.

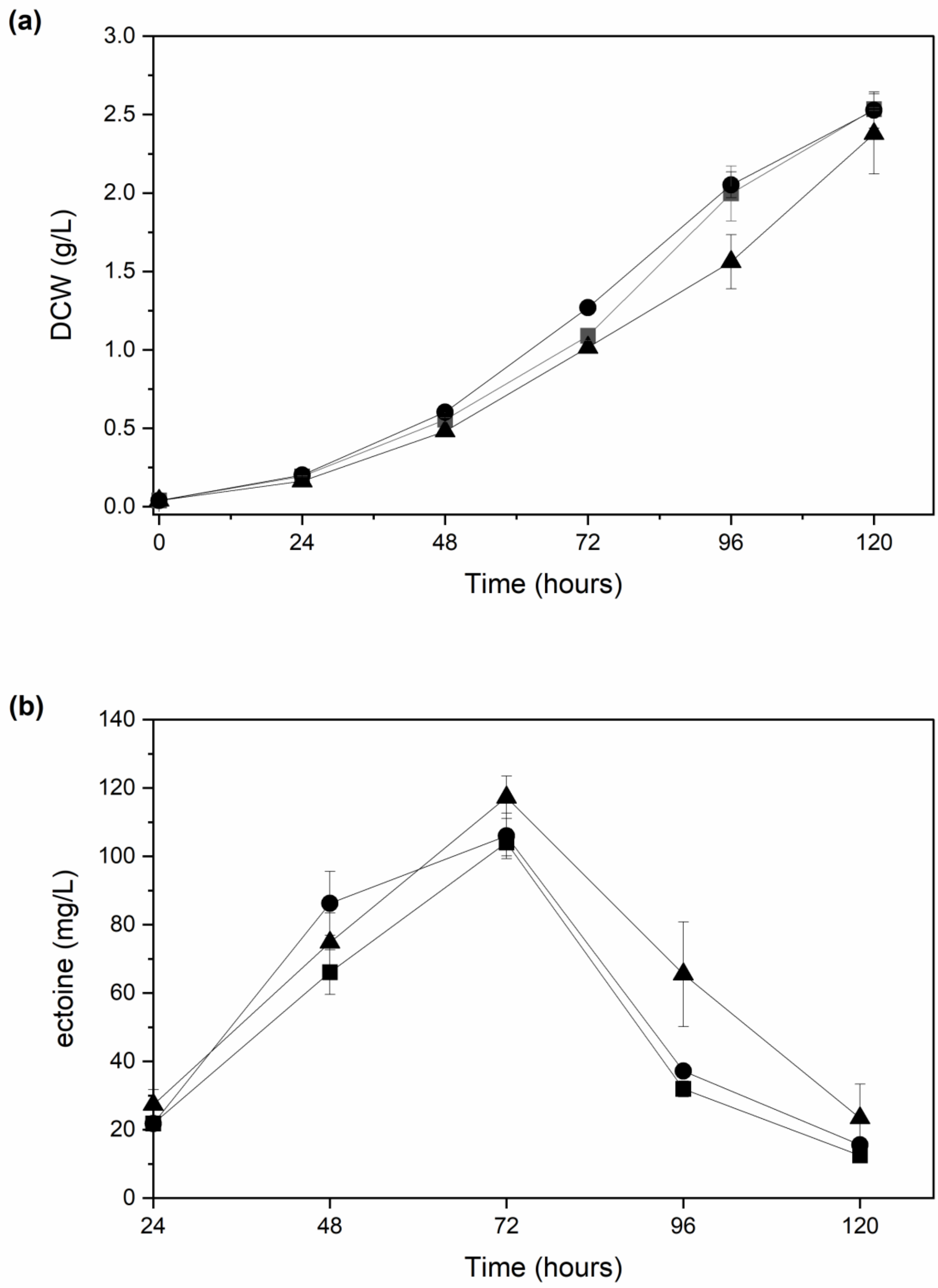

Figure S3. Effect of agitation speed on cell growth and ectoine production in *M. alcaliphilum* 20ZDP2. *M. alcaliphilum* 20ZDP2 was cultivated in a *Methylomicrobium* medium containing

6% NaCl and 0.05 μM of tungsten at 30 °C shaken at different agitation speed. (a) dry cell weight (DCW) and (b) ectoine production. The following symbols were used: 150 rpm (■), 230 rpm (●), and 300 rpm (▲). All experiments were performed in triplicate and the range of the raw data was within ± 5% of the average.

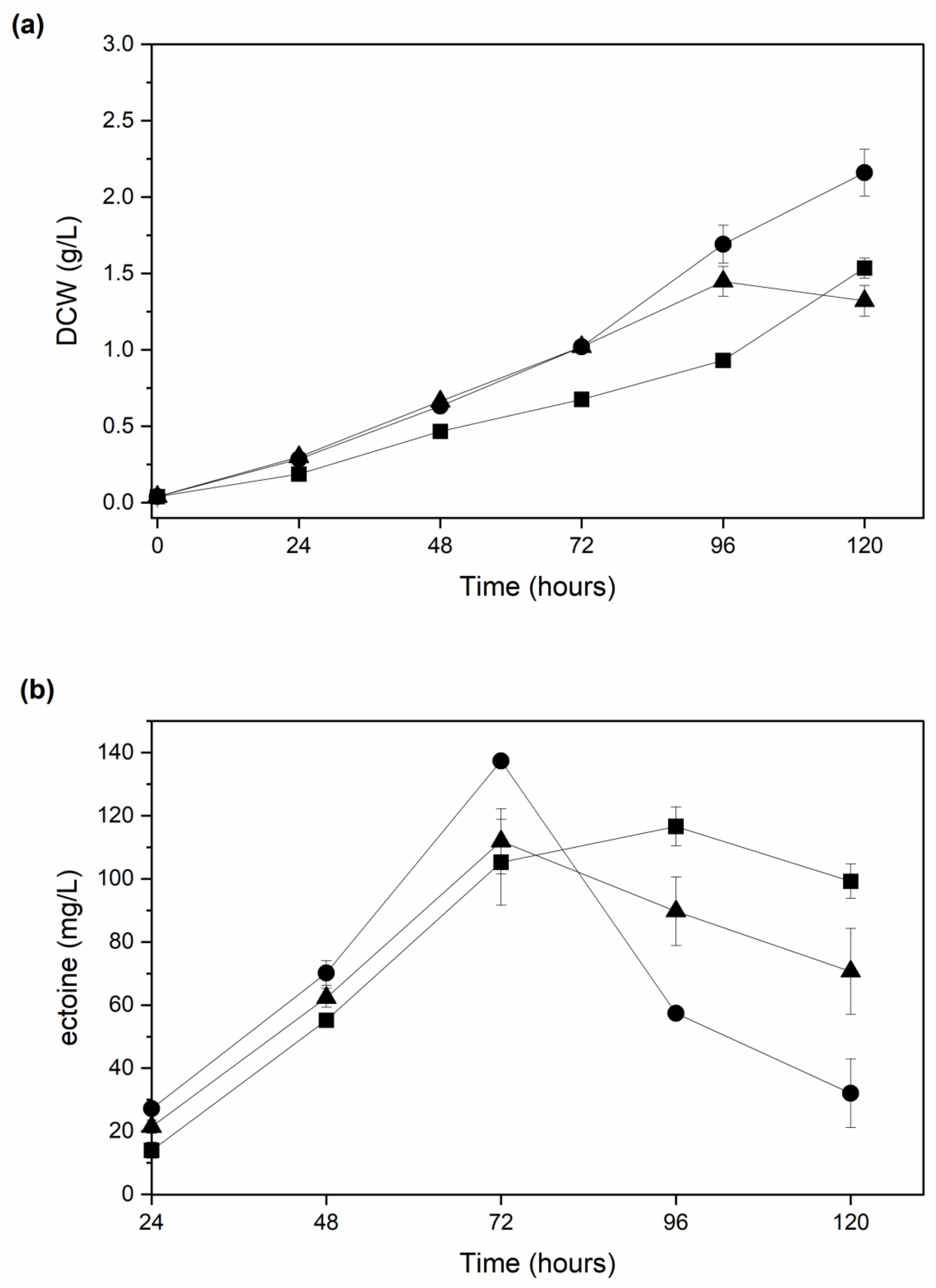

Figure S4. Effect of temperature on cell growth and ectoine production in *M. alcaliphilum* 20ZDP2. *M. alcaliphilum* 20ZDP2 was cultivated in a *Methylomicrobium* medium containing 6% NaCl and 0.05 μM of tungsten shaken 230 rpm at different temperature. (a) dry cell

weight (DCW) and (b) ectoine production. The following symbols were used: 25 ℃ (■), 30 ℃ (●), and 35 ℃ (▲). All experiments were performed in triplicate and the range of the raw data was within ± 5% of the average.
